# Indoor Air Pollutants and Health in the United Arab Emirates

**DOI:** 10.1289/ehp.1104090

**Published:** 2012-02-22

**Authors:** Karin B. Yeatts, Mohamed El-Sadig, David Leith, William Kalsbeek, Fatma Al-Maskari, David Couper, William E. Funk, Taoufik Zoubeidi, Ronna L. Chan, Chris B. Trent, Christopher A. Davidson, Maryanne G. Boundy, Maamoon M. Kassab, Mohamed Y. Hasan, Ivan Rusyn, Jacqueline MacDonald Gibson, Andrew F. Olshan

**Affiliations:** 1 Department of Epidemiology, Gillings School of Global Public Health, University of North Carolina–Chapel Hill, Chapel Hill, North Carolina, USA; 2 Department of Community Medicine, Faculty of Medicine and Health Sciences, United Arab Emirates University, Al Ain, United Arab Emirates; 3 Department of Environmental Sciences and Engineering; 4 Survey Research Unit, Department of Biostatistics, and; 5 Collaborative Studies Coordinating Center, Department of Biostatistics, University of North Carolina–Chapel Hill, Chapel Hill, North Carolina, USA; 6 Department of Anthropology, Northwestern University, Evanston, Illinois, USA; 7 National Bureau of Statistics, Abu Dhabi, United Arab Emirates

**Keywords:** asthma, incense, indoor air pollutants, neurologic, respiratory

## Abstract

Background: Comprehensive global data on the health effects of indoor air pollutants are lacking. There are few large population-based multi–air pollutant health assessments. Further, little is known about indoor air health risks in the Middle East, especially in countries undergoing rapid economic development.

Objectives: To provide multifactorial indoor air exposure and health data, we conducted a population-based study of indoor air pollution and health in the United Arab Emirates (UAE).

Methods: We conducted a cross-sectional study in a population-based sample of 628 households in the UAE. Indoor air pollutants [sulfur dioxide (SO_2_), nitrogen dioxide (NO_2_), hydrogen sulfide (H_2_S), formaldehyde (HCHO), carbon monoxide (CO), and particulate matter] were measured using passive samplers over a 7-day period. Health information was collected from 1,590 household members via in-person interviews.

Results: Participants in households with quantified SO_2_, NO_2_, and H_2_S (i.e., with measured concentrations above the limit of quantification) were twice as likely to report doctor-diagnosed asthma. Participants in homes with quantified SO_2_ were more likely to report wheezing symptoms {ever wheezing, prevalence odds ratio [POR] 1.79 [95% confidence interval (CI) 1.05, 3.05]; speech-limiting wheeze, POR 3.53 (95% CI: 1.06, 11.74)}. NO_2_ and H_2_S were similarly associated with wheezing symptoms. Quantified HCHO was associated with neurologic symptoms (difficulty concentrating POR 1.47; 95% CI: 1.02, 2.13). Burning incense daily was associated with increased headaches (POR 1.87; 95% CI: 1.09, 3.21), difficulty concentrating (POR 3.08; 95% CI: 1.70, 5.58), and forgetfulness (POR 2.68: 95% CI: 1.47, 4.89).

Conclusions: This study provides new information regarding potential health risks from pollutants commonly found in indoor environments in the UAE and other countries. Multipollutant exposure and health assessments in cohort studies are needed to better characterize health effects of indoor air pollutants.

Indoor air quality is increasingly recognized as a critical component of public health and as a subject for which comprehensive global data are lacking. Several regions of the world are undergoing an indoor air pollution risk transition, in which traditional health risks of household fuel combustion are subsiding and modern risks from airtight buildings and building materials are increasing (Zhang and Smith 2003). Little is known about the indoor health risk transition in the Middle East region and the Arab Gulf countries, many of which are in accelerated transition and development. Ambient air pollution in the Arabian Peninsula is characterized by dust storms, high levels of desert particulate matter (PM), transportation- and industry-related emissions, and meteorology-linked smog formation (Al-Rehaili 2002; Bu-Olayan and Thomas 2012; Engelbrecht et al. 2009). Ambient air pollutants may infiltrate and contribute to indoor air pollution that may also result from indoor combustion sources such as gas stoves and tobacco smoke, as well as traditional sources such as incense burning.

The United Arab Emirates (UAE) is a notable example of a country in accelerated transition. In the last 50 years, since the discovery of oil in the UAE, the country has transitioned rapidly from a nomadic and trading economy to an emerging industrialized nation with a per-capita gross domestic product ranked sixth in the world in 2011 (International Monetary Fund 2012). With this rapid economic growth has come large-scale infrastructure development, including new industries, transportation networks, and cities. One of the more dramatic changes that has occurred in the UAE has been the transition from naturally ventilated barasti (reed) huts and nomadic tents to tightly sealed, air-conditioned villas and apartments. This new built environment has brought the potential for exposure to pollutants that accumulate in these buildings. These indoor air pollutant concentrations and their related health effects have not been characterized.

To provide multifactorial indoor air exposure and health data, we conducted one of the first population-based studies of indoor air pollution and health in the Middle East. Our population-based cross-sectional household study in the UAE examined the effects of five gas pollutants [sulfur dioxide (SO_2_), nitrogen dioxide (NO_2_), hydrogen sulfide (H_2_S), formaldehyde (HCHO), and carbon monoxide (CO)], and three size fractions of PM [aerodynamic diameter of ≤ 2.5 µm (PM_2.5_), 2.5–10 µm (PM_2.5–10_), and ≤ 10 µm (PM_10_)] on the prevalence of respiratory and neurologic symptoms in children and adults.

These pollutants include indoor pollutants emitted from common indoor air sources that have known or suspected health effects (NO_2_, H_2_S, HCHO, CO, PM), and markers of infiltration from outdoor air sources (SO_2_, H_2_S, PM). SO_2_, NO_2_, H_2_S, HCHO, and PM have been linked with respiratory disease symptoms; HCHO and CO have been associated with neurologic symptoms (Bernstein et al. 2008; Guidotti 1996; Johns and Linn 2011; McGwin et al. 2011; Townsend and Maynard 2002; Wilbur et al. 1999).

## Materials and Methods

*Study design and population.* We conducted a population-based cross-sectional study to explore the relationship between indoor air pollutants and respiratory and neurologic symptoms in the UAE. A nationally representative sample of 628 Emirati households from the UAE population was recruited following a two-stage cluster sample design that included stratification by geographic area and population density (Lohr 2010). In collaboration with the UAE National Bureau of Statistics (formerly the Ministry of Economy), we used the most recent 2005 UAE Census data, with a master sample update from May 2008. Sufficient numbers of households were selected using a two-stage cluster sampling strategy to ensure the study sample represented Emirati households in rural and urban areas of all seven emirates (Abu Dhabi, Dubai, Sharjah, Ajman, Umm al Qwain, Ras al Khaimah, and Fujairah). The 14 strata were divided into clusters (primary sampling units, or PSUs), each of which consisted of a census enumeration area (in urban areas) or a village (in rural areas). Data on the size and density of the national population by emirate are provided in Supplemental Material, Table 1 (http://dx.doi.org/10.1289/ehp.1104090). One hundred twenty PSUs were selected from the 14 strata, and a simple random sample of seven to eight households was chosen from each PSU. These households were then located and approached in person by Arabic-speaking interviewers, and the families were invited to participate. The study protocol was approved by the institutional reviews boards of both the University of North Carolina–Chapel Hill (UNC) and the UAE University Faculty of Medicine. In accordance with the cultural guidelines recommended by our collaborators at UAE University, written informed consent was obtained from the head of household for all family members residing in the home. Household participation in the study was declined if the head of household did not provide consent for the household members.

**Table 1:** Demographic, household, and environmental exposure variables (total n = 1,590).

Variable	na	Wtd%b
Demographic variable				
Age category				
Adults (19–50 years)		1,007		59.4
Adolescents (11–18 years)		330		27.1
Children (6–10 years)		253		13.6
Sex				
Female		811		51.8
Male		779		48.1
Urban/rural				
Urban		878		57.6
Rural		712		42.4
Head of household education level				
College to postgraduate		488		28.82
Secondary		568		35.64
Preparatory or middle school		231		15.61
Primary school		158		9.96
None/cannot write/cannot read		136		9.59
Birthplace of head of household				
Nomadic settlement in the desert		179		13.09
Rural village (up to 10,000 people)		263		16.48
Small town (10,000–24,999 people)		286		15.48
Large town (25,000–49,999 people)		90		4.77
City (50,000–199,999 people)		243		17.24
Large city (Abu Dhabi, Dubai)		426		25.9
Don’t know		15		0.69
Household and environmental exposure variable				
Type of household building				
Villa		699		49.25
Shabia (governmental housing)		492		32.29
Flat/apartment		268		10.42
Palace		15		0.81
Other		30		1.11
Missing		86		6.08
Frequency of incense used in home in a typical week				
Never		105		5.44
Once		231		11.94
2–5 times per week		529		30.92
Daily		586		43.54
Missing		139		8.13
Type of cooking equipment				
Gas		971		64.09
Electric		98		5.64
Gas and electric		382		22.12
Missing		139		8.1
Kitchen configuration and gas cooking equipment				
Attached to main living area and gas stove exclusively		315		17.08
Separate building (gas and/or electric) or attached electric stove		1,136		74.78
Missing		139		8.13
aNumber of individuals; household-level data were assigned to each individual within a household. bPercentages statistically weighted by participant-level weights.				

At the first household visit, the head of household was interviewed. Household family members were selected for individual interviews during the second visit, based on an algorithm that allowed equal probability of selection for each member in a household within four age/sex categories: adult male (19–50 years of age), adult female (19–50 years of age), adolescent (11–18 years of age), and child (6–10 years of age). For all interviews involving children, responses were obtained from the mother.

*Study procedures.* We collected data during an 8-month period from October 2009 through May 2010. Data collection required two home visits. During the first visit, interviewers obtained informed consent, obtained a list of the family members residing at the address, and deployed air-monitoring equipment. A three-member interviewer team made a second visit approximately 7 days later. During the second visit, the interviewer team took readings from the equipment, retrieved the air-monitoring equipment, and interviewed the selected family members regarding household- and individual-level information on current and past medical histories, respiratory symptoms, housing characteristics, potential environmental household exposures, and behavioral and lifestyle factors such as smoking and physical activity. The head of household was interviewed regarding the household socioeconomic status, residential history, and environmental exposures.

All interviews were conducted by trained Arabic-speaking field interviewers, who entered each participant’s response directly into the data management system during the computer-assisted personal interviews. All data were uploaded weekly and securely transmitted to the Collaborative Studies Coordinating Center (CSCC) at UNC for data processing and quality control checks.

*Exposure and health assessment.* Indoor air pollutant measurements. Five gaseous pollutants and three sizes of PM (PM_2.5_, PM_2.5–10_, and PM_10_) were measured passively indoors over a 7-day period. SO_2_, NO_2_, H_2_S, HCHO, and CO were measured using colorimetric diffusion tubes (Gastec Corp., Kanagawa, Japan) (Nash and Leith 2010). Passive aerosol samplers were used to measure PM_2.5_, PM_2.5–10_, and PM_10_ (Wagner and Leith 2001).

During the first household visit, air-sampling equipment was inserted into a sampling block, affixed to a tripod at a standardized height of 1.3 m, and covered with a protective metal cage. The sampling equipment was then deployed in a common living room shared by both male and female members of the household. In a small proportion of homes, the samplers were deployed without the tripod because of spatial constraints and/or owner concerns.

At the second visit, the diffusion tubes were read independently by two field team interviewers who entered a consensus reading into the computer. Time-weighted averages for the gaseous concentrations were later determined using pollutant-specific algorithms (Nash and Leith 2010). PM samplers were collected and sent to RJ Lee Group, Inc. (Monroeville, PA, USA) for analysis. For quality control, a randomly selected duplicate diffusion tube was deployed and read in each household. In 10% of the households, duplicate PM samplers were deployed. The limits of quantification for SO_2_, NO_2_, H_2_S, and HCHO were 0.010, 0.006, 0.060, and 0.006 ppm, respectively.

Nash and Leith (2010) tested a range of concentrations and potential interferences to investigate the ability of the Gastec tubes to passively measure low gaseous concentrations over a 1-week period. They also performed extensive quality control tests on random samples from different lots of tubes to assess variance. In the present study, the field interviewers read the diffusion tubes directly in the field and, therefore, were not blinded as to the identity of the duplicates. The average relative standard deviations of the duplicates were 8%, 4%, 5%, 5%, and 11% for SO_2_, NO_2_, H_2_S, HCHO, and CO, respectively (Funk WE, unpublished data). For the PM samplers, the averages of the relative standard deviations for the 33 duplicate PM samples were 20%, 16%, and 15% for PM_2.5_, PM_2.5–10_, and PM_10_, respectively (Funk WE, unpublished data). No particles were detected on the blank PM samplers.

Health and exposure questionnaire data. Medical history and smoking questions were adapted from both the National Health and Nutrition Examination Survey (NHANES) [Centers for Disease Control and Prevention (CDC) 2010c] and the National Health Interview Survey (CDC 2010b). Respiratory symptoms and atopy were assessed using the International Study of Asthma and Allergies in Childhood (Pearce et al. 2007) and Behavioral Risk Factor Surveillance System questions (CDC 2010a). In addition, clusters of respiratory and neurological symptoms were adapted from Douwes et al. (2001).

Questions for the environmental household exposure section of the questionnaire were adapted from the Relationship of Indoor, Outdoor and Personal Air Study survey (Weisel et al. 2005) and the UAE Health and Lifestyle Survey (UAEHALS) 2000 (Badrinath et al. 2002). Input on culture-specific household activities and cultural appropriateness was solicited from UAE University faculty and additional UAE colleagues. All survey questions were translated into Arabic, then translated back into English, and reviewed by a UAE University researcher to ensure the translations were accurate, culturally appropriate, and reflected local terminology.

Household-level data were assigned to each individual within a household. For statistical analysis, the incense variable responses were collapsed into three categories: none or once a week, two to five times a week, and daily burning in a typical week, with the referent group as none/once a week. In the exposure–disease analyses, we present comparisons for daily incense burning compared with burning once a week or less. Household tobacco smoke was defined as a dichotomous exposure: any tobacco smoke exposure versus no tobacco smoke exposure in the household in a typical week.

*Quality control and quality assurance.* The data management system (DMS), developed by researchers at the UNC CSCC, provided several major functions: *a*) entry, editing, and updating of data in the field; *b*) transfer of data to the CSCC via the Internet; and *c*) maintenance of an up-to-date status of household recruitment and interview completions. Data quality checks were operational during all processes of the data entry, transfer, tracking, and analyses.

*Statistical analysis.* Initially, we calculated univariate means and prevalence estimates for the indoor air quality measurement, demographic, household exposure, and symptom outcome variables. Gas concentrations below the limit of quantification were assumed to be zero in the calculation of the air pollutant means. Because of the highly skewed distributions of SO_2_, NO_2_, H_2_S, and HCHO, we created dichotomous exposure variables defined as quantified gas concentrations versus gas concentrations below the limit of quantification. We used multiple logistic regression to estimate adjusted prevalence odds ratios (PORs) for the odds of each outcome in the exposed population compared with the nonexposed population. We adjusted for confounders identified by using a 10% change in the effect estimate criterion, including sex, urban/rural area, age group (adult, adolescent, child), and household tobacco smoke exposure (any/none). Other potential confounders not included in final models were education level of the head of household, mold in the household, pet ownership, parental allergies, and parental asthma. All analyses were conducted with SAS software, version 8.2 (SAS Institute Inc., Cary, NC, USA).

We applied sampling weights using SAS survey procedures. Household sampling weights (W*_j_*) were provided by the UAE National Bureau of Statistics. Participant sampling weights for each of four age/sex participant categories (adult females, adult males, adolescents, and children) were calculated as W*_j_*, × f*_ij_*, where f*_ij_* was the total number of individuals in the *i*th household in the *j*th PSU. The sampling weights were winsorized to 1,000 to reduce the influence of extreme weighted values. Winsorizing is a statistical technique in which an extreme value is set to a less extreme value in order to reduce the effect of the extreme value. The SAS survey procedures also account for within-household clustering.

To assess the potential influence of outliers on the exposure–disease associations, we conducted a sensitivity analysis excluding the highest 1% and the highest 5% of the air pollutant measurements. Results were consistent for SO_2,_ NO_2_, HCHO, CO, and the three PM size fractions. Although the reduced data set associations were less precise than the full data set results, the associations were generally consistent with respect to the direction and magnitude of the full data set associations.

## Results

Of the 827 households invited to participate, 628 agreed, yielding a household response rate of 75%. Our study sample demographics reflected the general population of UAE national households. The distribution of sample households by emirate was very similar to that of the UAE 2005 Census distribution [see Supplemental Material, Table 2 (http://dx.doi.org/10.1289/ehp.1104090)]. For example, 40% of our study sample households lived in Abu Dhabi emirate compared with 42% nationally.

**Table 2:** Measured indoor air pollutant concentrations^a^ and dichotomized gas exposure variables.

Air pollutant concentrations	Limit of quantificationc	Percentile							Maximum value	Quantifed gasd	Dichotomized gas exposure variables						
		nb	Median	75	90	95	99	ppm			µg/m3	n	Wtd%e
SO2 (ppm)		1,586		< 0.010c		0.010c		0.014		0.042		0.061		0.454		0.507		Any		0.010–0.507		26.2–1,327		548		29.85
																		None		< 0.010		< 26.2		1,038		70.01
NO2 (ppm)		1,587		< 0.006c		0.006c		< 0.006c		< 0.006		0.012		0.047		0.048		Any		0.006–0.048		11.3–90.3		186		9.39
																		None		< 0.006		< 11.3		1,401		90.48
H2S (ppm)		1,587		< 0.060c		0.060c		< 0.060c		0.09		0.150		0.337		1.098		Any		0.060–1.098		83.4–1,527		256		12.62
																		None		< 0.060		< 83.4		1,331		87.25
HCHO (ppm)		1,587		< 0.006c		0.006c		0.007		0.034		0.048		0.093		0.137		Any		0.006–0.137		7.37–168.2		535		28.80
																		None		< 0.006		< 7.37		1,052		71.07
CO (ppm)		1,586		0.761		0.30		1.039		1.544		1.84		4.74		5.81										
PM2.5 (µg/m3)		1,463		6.20		NA		9.62		14.92		19.14		34.66		167.26										
PM2.5–10 (µg/m3)		1,463		36.95		NA		54.10		78.78		100.76		213.19		264.81										
PM10 (µg/m3)		1,463		43.98		NA		62.10		92.07		121.64		246.43		421.86										
NA, not applicable. aHousehold air pollutant concentrations weighted with participant-level sampling weights (units are parts per million for gases and micrograms per cubic meter for PM). bNumber of individuals; household-level data were assigned to each individual within a household. cAir pollutant limit of quantification (Funk WE, unpublished data). dBased on limit of quantification; conversions to micrograms per cubic meter use 25°C, 1 atm. ePercentages statistically weighted by participant-level weights.																										

The average household size was 6, with a range of 1–25 family members. Individual participation rates from the five age/sex categories were 94% (585/621) for heads of household, 77% (484/627) for adult males, 82% (523/638) for adult females, 73% (330/452) for adolescents, and 88% (253/287) for children. Approximately 60% of the source population included in the analysis were 19–50 years of age, and 60% lived in urban areas (Table 1).

The demographic data provide evidence of a society in transition (Table 1). A large percentage of the heads of household was born in cities (43%), and 29% had a college to postgraduate education. On the other hand, 13% of the heads of household were born in nomadic settlements, and 10% could not read or write. Thirty-two percent of the study population lived in shabias [governmental housing, provided in the 1970s to encourage the settlement of nomadic tribes (Table 1)], whereas 49% of the source population lived in villas and 79% lived in homes they owned (Table 1). Households owned a median of three computers, and three cars per household (data not shown).

*Environmental exposures.* Secondhand tobacco smoke, incense, gas stoves. Nineteen percent of the source population was exposed to secondhand tobacco smoke in their homes, and 86% were exposed to burning incense at least once a week, with 44% exposed every day (Table 1). Although gas stoves were exclusively used in 64% of homes in the source population, only 17% of the study population lived in households where gas was used exclusively and the kitchen was attached to the main residence.

Measured indoor air pollutant concentrations. Table 2 provides the distribution of the measured indoor household air pollutant concentrations for the five gaseous species and their respective limits of quantification. These data are participant-weighted concentrations (statistically weighted by the participant sample weights and the number of participants per household) and represent the study population’s exposures. The measured SO_2_, NO_2_, H_2_S, and HCHO concentrations were skewed, with median concentrations below the limit of quantification. For both NO_2_ and H_2_S, we estimated that 75% of the source population had household exposures below their respective limits of quantification (Table 2). Household concentrations of CO and all size fractions of PM were low compared with international guidelines (World Health Organization 2000); high concentrations of PM were reported in a minority of homes (Funk WE, unpublished data). Given the skewed nature of the gas concentration data, we dichotomized our gas pollutant exposures into quantified gas concentrations versus gas concentrations below the limit of quantification (Table 2). Conversions from units of parts per million to micrograms per cubic meter were calculated at 25°C, 1 atmosphere (atm).

After adjusting with population sample weights, we estimated that 30% of the source population lived in homes with quantified SO_2_, and 29% lived in homes with quantified HCHO. Smaller proportions, 9% and 12%, were exposed to quantified household concentrations of NO_2_ and H_2_S, respectively. These four gaseous pollutants were modestly correlated with each other. SO_2_ was correlated with NO_2_ (Pearson’s correlation coefficient, *r* = 0.68), H_2_S (*r* = 0.59), and HCHO (*r* = 0.63). The correlation coefficients for CO were lower and ranged from the *r* = 0.18 for NO_2_ and HCHO to the *r* = 0.36 for SO_2_. The PM fractions were highly correlated with each other (PM_10_ × PM_2.5–10_, *r* = 0.99; PM_10_ × PM_2.5_, *r* = 0.82; PM_2.5–10_ × PM_2.5_, *r* = 0.72), but not with the gases [see Supplemental Material, Table 3 (http://dx.doi.org/10.1289/ehp.1104090)].

**Table 3:** Frequency of wheezing symptoms and doctor-diagnosed asthma in study participants.

Wheezing symptom and doctor-diagnosed asthmaa	Value	n	Wtd%b	95% CI
Ever had wheezing		Yes		197		13.12		11.5, 14.8
		No		1,374		85.42		83.7, 87.2
		Missing		19		1.45		0.9, 2.0
Wheezing in last 12 monthsc		Yes		147		9.17		7.8, 10.6
		No		49		3.94		3.0, 4.9
		Missing		1,394		86.89		85.2, 88.5
Wheezing in last 4 weeksc		Yes		99		6.08		4.9, 7.2
		No		48		3.10		2.2, 3.9
		Missing		1,443		90.83		89.4, 92.2
Wheezing limited speech to 1 or 2 words between breathsc		Yes		43		3.90		2.9, 4.8
		No		102		5.19		4.1, 6.3
		Missing		1,445		90.91		89.5, 92.3
Ever doctor-diagnosed asthma		Yes		142		8.22		6.9, 9.6
		No		1,432		90.29		88.8, 91.7
		Missing		16		1.48		0.9, 2.1
aIncludes adults, adolescents, and children. bPercentages statistically weighted by participant-level weights. cHigh numbers of missing are due to skip patterns for these symptoms.								

SO_2_ was not significantly associated with either gas stoves in kitchens attached to the main residence or tobacco smoke reported in the home (data not shown). Quantified SO_2_ was more than twice as likely [odds ratio (OR) 2.60; 95% confidence interval (CI): 1.58, 4.27) and quantified H_2_S (OR 3.34; 95% CI: 1.49, 7.47) and HCHO (OR 3.10; 95% CI: 1.84, 5.22) were three times as likely in households burning incense two or more times a week compared with homes where incense burned once a week or less. Incense burning (as an ordinal variable with one or less times/week, two to five times/week, daily as categories) was associated with increasing PM_2.5_, PM_2.5–10_, and PM_10_ quartile concentration [Cochran–Mantel–Haenszel Statistic, general association (ordinal scales for both variables)] with *p*-values of 0.020, 0.0005, and 0.0009, respectively. Quantified NO_2_ was twice as likely (OR 2.13; 95% CI: 1.15, 3.94) in households where a gas stove was located in a kitchen attached to the main living area as in households where the kitchen was in a separate building. Tobacco smoking in the household was associated with measured CO (*p*-value 0.0001), but not with other gaseous or particulate pollutants (data not shown).

*Health outcomes.* After adjusting for sampling weights, 13%, 9%, and 8% of the population reported ever wheezing, current wheezing (wheezing in the last 12 months), and ever having doctor-diagnosed asthma, respectively. Twelve percent of the population reported chest tightness/difficulty breathing [see Supplemental Material, Table 4 (http://dx.doi.org/10.1289/ehp.1104090)], and 4% reported speech-limiting wheeze, the most severe asthma symptom (Table 3). The frequencies of other respiratory symptoms (e.g., cough, shortness of breath) are presented in Supplemental Material, Table 4. Headache in the last 12 months was the most common neurologic symptom (46%), and dizziness (12%) was the least common (see Supplemental Material, Table 5).

**Figure 1:**
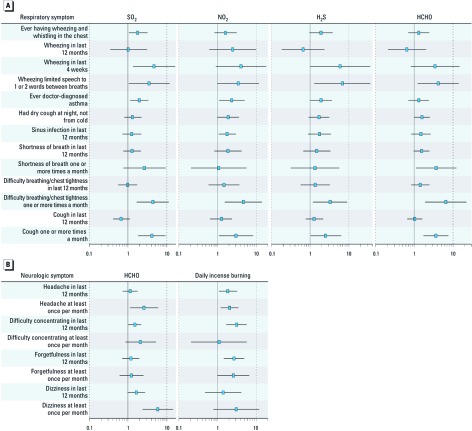
Forest plots showing associations between indoor air pollutants and respiratory (*A*) and neurologic (*B*) symptoms. (*A*) Forest plots with the adjusted PORs of respiratory symptoms, doctor-diagnosed asthma versus quantified household SO_2_, NO_2_, H_2_S, and HCHO concentrations. Error bars represent 95% CIs. Models are adjusted for sex, urban/rural area, age group, and household tobacco smoke exposure. (*B*) Forest plots with the adjusted PORs of neurologic symptoms versus quantified household HCHO concentrations, and daily indoor incense burning. Error bars represent 95% CIs. Models are adjusted for sex, urban/rural area, age group, and household tobacco smoke exposure.

*Indoor air exposures and symptom associations.* The patterns of association with respiratory symptoms were similar for SO_2_, NO_2_, H_2_S, and HCHO [Figure 1A; see also Supplemental Material, Table 6 (http://dx.doi.org/10.1289/ehp.1104090)]. Symptoms of ever wheezing, wheezing in the last 4 weeks, speech-limiting wheezing in the last 12 months, and doctor-diagnosed asthma were all significantly associated with quantified household concentrations of SO_2_ and H_2_S. Family members with quantified SO_2_ in their homes were 1.95 times as likely to have doctor-diagnosed asthma (adjusted POR 1.95; 95% CI: 1.13, 3.36) as family members of homes with no quantified SO_2_. Similarly, those with quantified SO_2_ were more likely to report ever wheezing (POR 1.79; 95% CI: 1.05, 3.05), wheezing in the last 4 weeks (POR 4.63; 95% CI: 1.33, 16.19), and speech-limiting wheeze in the last 12 months (POR 3.53; 95% CI: 1.06, 11.74). Associations of these outcomes with NO_2_, H_2_S, and HCHO were similar, although not all were statistically significant. We also found positive associations for SO_2_, NO_2_, H_2_S, and HCHO with other respiratory symptoms such as shortness of breath, difficulty breathing, chest tightness, and cough. We did not find consistent significant associations of increased respiratory symptoms with gas stoves in kitchens attached to the main residence or gas stoves in general, but the majority of associations with gas stoves were positive (see Supplemental Material, Figure 1).

Tobacco smoking in the household was associated with increased wheezing in the last 12 months (POR 3.49; 95% CI: 1.18, 10.35) and ever having doctor-diagnosed asthma (POR 1.96: 95% CI: 1.06, 3.63) as well as dry cough at night (not from cold), shortness of breath, difficulty breathing/chest tightness in the last 12 months, and coughing one or more times a month [see Supplemental Material, Figure 1 (http://dx.doi.org/10.1289/ehp.1104090)]. Although other associations between indoor tobacco smoke and respiratory symptoms were not statistically significant, they were positive and above the null. No consistent pattern of associations was found between respiratory symptoms and incense use (see Supplemental Material, Figure 1), the three size fractions of PM, or CO concentrations (see Supplemental Material, Figure 2).

In households with quantified HCHO concentrations, participants reported an increase in difficulty concentrating (POR 1.47; 95% CI; 1.02, 2.13) and dizziness in the last 12 months (POR 1.64; 95% CI: 0.97, 2.77) [Figure 1B and Supplemental Material, Table 7 (http://dx.doi.org/10.1289/ehp.1104090)]. Quantified HCHO was also associated with headache (POR 2.57; 95% CI: 1.11, 5.97) and dizziness (POR 5.85; 95% CI: 2.35, 14.56) at least once a month or more. Forgetfulness was not significantly associated with HCHO; although the association was positive, it was close to the null. These results should be interpreted with caution given the small numbers.

Participants who lived in households where incense was burned daily were two to four times as likely to report headaches (POR 1.87; 95% CI: 1.09, 3.21), difficulty concentrating (POR 3.08; 95% CI: 1.70, 5.58), and forgetfulness (POR 2.68; 95% CI: 1.47, 4.89) in the last 12 months, as those living in households in which incense was burned once a week or not at all [Figure 1B and Supplemental Material, Table 7 (http://dx.doi.org/10.1289/ehp.1104090)]. Daily exposure to incense was also associated with headaches (POR 2.05; 95% CI: 1.22, 3.45) and forgetfulness (POR 2.64; 95% CI: 1.02, 6.78) one or more times a month.

## Discussion

Our study is one of the few large international population-based multi–air pollutant health assessments and one of the first assessments of the relationship between indoor air pollutant exposures and health outcomes in a region where environmental health studies are rare (Adams et al. 2011). We report a unique profile of pollution sources in this population, including frequent incense burning and separated kitchens with gas stoves; these sources should be considered in future indoor air pollution research in this region.

Quantified indoor concentrations of SO_2_, NO_2_, and H_2_S were associated with respiratory symptom prevalence, symptom frequency, and doctor-diagnosed asthma. Quantified HCHO was associated with respiratory symptoms. We found evidence of increased neurologic symptoms among participants who had quantified HCHO concentrations in their homes and among participants who burned incense two or more times a week. We did not observe associations of indoor PM or CO concentrations with respiratory or neurologic symptoms.

We identified few studies of indoor SO_2_ measurements with which to compare our findings. Our study participants with quantified concentrations of SO_2_ in their households (range 0.010–0.507 ppm, or 26.2–1327 µg/m^3^) were 1.79–4.6 times as likely to report asthmatic symptoms of ever-wheeze, wheeze in the last 4 weeks, speech-limiting wheeze in the last year, and doctor-diagnosed asthma. Our household SO_2_ concentration levels are comparable with those reported by Zhao et al. (2008; i.e., 60–641.1 µg/m^3^) based on 7-day passive diffusion monitoring in 34 school classrooms in a coal-burning region of China (Zhao et al. 2008). In that study, a 100-µg/m^3^ increase in SO_2_ was associated with wheezing or whistling in the chest (OR 1.18; 95% CI: 1.03, 1.35) and nocturnal attacks of breathlessness (OR 1.28; 95% CI: 1.02, 1.59). The magnitudes of these relative risk estimates are consistent with our findings.

Studies on the health effects of outdoor SO_2_ and respiratory symptoms, most of which have classified exposure using central site monitoring data, have been mixed. An analysis of National Health Information Survey data for 34,073 children (Akinbami et al. 2010) did not find evidence of increased asthma attacks with SO_2_ concentrations ranging from 0.0001 to 0.0166 ppm. Similarly, Ramadour et al. (2000) found no evidence of health effects in a cross-sectional study of 2,445 children with SO_2_ outdoor concentrations ranging from 17.3 to 57.4 µg/m^3^ (0.007–0.022 ppm). In contrast, Pikhart et al. (2000) found increased wheezing (OR 1.17; 95% CI: 1.01, 1.35) per 10-µg/m^3^ increase in SO_2_ in a cross-sectional study of 3,045 children.

Our 0.006–0.048 ppm (11.3–90.3 µg/m^3^) range of quantified NO_2_ was associated with respiratory symptoms in study participants, including speech-limited wheezing (OR 3.48; 95% CI: 0.99, 12.22) and dry cough at night not due to a cold (OR 1.90; 95% CI: 1.00, 3.60). The range of exposure in our study was slightly wider than but comparable with NO_2_ concentrations of 15.5–61.6 µg/m^3^ (0.008–0.033 ppm) reported in a school-based cross-sectional study of 1,480 students in China (Zhao et al. 2008), 0.0005–0.480 ppm in bedrooms of 469 children from eight U.S. cities (Kattan et al. 2007), and 0.0029–0.394 ppm in bedrooms of 150 children with asthma in Baltimore, Maryland (Hansel et al. 2008). Associations between NO_2_ and respiratory symptoms reported by these studies ranged from an incidence rate ratio (IRR) of 1.15 (95% CI: 1.05, 1.25) per 20-ppb increase in NO_2_ (Hansel et al. 2008) to an OR of 1.45 (95% CI: 1.00, 2.08) for nocturnal attacks of breathlessness per 10-µg/m^3^ increase in NO_2_ (Zhao et al. 2008), to an OR of 1.75 for increased asthma symptoms in nonatopic children (95% CI: 1.1, 2.78) (Kattan et al. 2007).

In our data, quantified H_2_S concentrations were associated with increased respiratory symptoms (e.g., OR 6.03; 95% CI: 1.00, 36.23 for wheezing symptoms in the last 4 weeks) and doctor-diagnosed asthma (OR 1.9; 95% CI 1.00, 3.60), albeit with wide CIs. However, these estimates should be interpreted with caution and require further investigation as to their plausibility. The upper value of the H_2_S range is substantially above the odor threshold, and it would have been difficult for family members to spend sustained amounts of time in a room with concentrations that high. The measured H_2_S concentrations might be the result of a cross-reaction or interference of pollutants other than those we measured.

We found no associations between indoor PM and respiratory symptoms. However, associations between PM and respiratory symptoms have been well documented in the scientific literature [U.S. Environmental Protection Agency (EPA) 2009]. In our study, there was evidence that outdoor PM may have had an important contribution to indoor PM (Funk WE, unpublished data). Outdoor PM in the UAE is substantially influenced by the desert environment and frequent dust storms throughout the year. Atmospheric models suggest that haboobs (i.e., dust storms) contribute up to 30% of the regional scale (1,000 × 1,000 km) total dust production in the southeastern Arabian Peninsula (Miller et al. 2008). Currently little data exist on the effects of ambient dust storm and non-urban windblown crustal particles on respiratory disease, although there are a growing number of studies examining dust storms and cardiovascular disease (U.S. EPA 2009). Active research and discussion continue with regard to the human health impacts of PM size, composition, and source (Brunekreef and Forsberg 2005; U.S. EPA 2009).

We found increased respiratory symptoms and doctor-diagnosed asthma prevalence in participants with household tobacco exposure, in agreement with the existing evidence on the respiratory effects of environmental tobacco smoke.

Incense burning, an anthropogenic source of indoor air pollutants, was ubiquitous: 86% of households burned incense indoors at least once a week. Existing research on incense has focused primarily on incense in East Asia, which differs from incense used in the Arabian Peninsula, including bakhour, a paste made from sandalwood tree resin mixed with other natural oils and substances (Wahab and Mostafa 2007) that traditionally is used by women, and oud (agarwood), which is used by both men and women. Jetter et al. (2002) found that particle and gas emissions varied substantially among 23 incense types used in Southeast Asia and India, with PM_2.5_ emissions from 7 to 202 mg/hr. Rocks/charcoal produced larger particles than other incense types, and emissions of SO_2_, NO, and CO also varied by incense.

Studies on the respiratory effects of incense in the Arabian peninsula are limited. A small case–control study of 100 Qatari children with asthma and 100 healthy controls (Wahab and Mostafa 2007) found that the prevalence of exposure to incense was significantly higher in asthmatic children (bakhour 80%, oud 65%, and frankincense 69%) compared with nonasthmatic children (66%, 51%, and 52%, respectively). In neighboring Oman, Al-Rawas et al. (2009) found that bakhour use at home was not associated with asthma in a cross-sectional study of 2,441 school children.

We did not find increased respiratory symptoms in participants who burned incense indoors more frequently. Instead, the presence of a person with asthma in the family was associated with reduced incense burning in the household. Families with an asthmatic child or adult may burn incense less frequently if incense exacerbates their asthma, but we could not evaluate this hypothesis in our study with our study design.

Incense burning two or more times a week was associated with headaches, forgetfulness, and loss of concentration. Although literature on the potential neurologic effects of incense is limited, Iijima et al. (2009) examined the effect of incense on brain function in 10 subjects using electroencephalograms. Their findings suggested that incense may enhance cortical activities and inhibitory motor response processing. We also found positive associations between neurologic symptoms and HCHO, which was three times as likely to be quantified in homes that burned incense two or more times a week than in homes that burned incense less than once a week. More investigation is needed to better understand both the constituents of incense and their possible neurologic and respiratory effects.

Our cross-sectional study design has both limitations and strengths. Because exposures and outcomes were assessed at a single point in time, we cannot determine whether the exposure preceded the outcomes or examine changes over time. Recall bias is unlikely, because air pollutant concentrations were measured in the home and not self-reported, and reported indoor air pollution source data were collected from the head of household.

There are limitations of our exposure measurement. We measured concentrations only in a common living area and assigned the same exposures to all participants in a household. In addition, we did not measure exposures at workplaces or schools or account for variation in the amount of time spent at home. Our crude metric of air pollutant exposure indicates only the likely presence or absence of the pollutant in the home. There is likely measurement error with regard to time frames for both the pollutant exposures and symptom outcomes. These sources of misclassification are unlikely to be related to the actual exposures or the outcomes and therefore would be expected to attenuate the exposure–disease associations toward the null value (Jurek et al. 2008).

Despite the design and measurement limitations, the cross-sectional design with 1-week average air pollutant measurements per household allowed us to rapidly assess indoor air pollutant exposures and their health effects in a country and region where little was known about the distribution of indoor air pollutants in homes and their health effects. Strengths of this study include a population-based sampling frame that allowed us to derive respiratory and neurologic symptom prevalence estimates that are nationally representative of UAE citizens in rural and urban areas across all seven emirates. The frequency of incense use, gas stoves, and passive tobacco smoke exposure, as well as our gas and PM measurements, are also nationally representative estimates. In general, the published epidemiologic and toxicological research support a biologically plausible association between SO_2_, NO_2_, H_2_S, and HCHO and respiratory outcomes (Bernstein et al. 2008; Guidotti 1996; Johns and Linn 2011); likewise, HCHO has been biologically associated with neurologic symptoms (Wilbur et al. 1999).

Our comprehensive multipollutant assessment of five gases and three PM size fractions allowed us to examine covariation among the pollutants and the differences of these air pollutants among the households. In the field, the diffusion tubes proved to be an unobtrusive and inexpensive method for measuring gas concentrations. Our estimates of household indoor air pollutant exposures are derived from an integrative measurement over 1 week that did not capture temporal or spatial variations of pollutant concentrations within the household. Nonetheless, we detected biologically plausible associations with respiratory and neurologic symptoms.

## Conclusions

We found a wide range of indoor air pollutant exposures in a large population-based sample. For the small proportion of participants living in homes with quantified SO_2_, NO_2_, and H_2_S concentrations, respiratory symptoms and doctor-diagnosed asthma were more likely than when compared with participants living in households with no quantified concentrations. Participants with quantified HCHO in their household reported increased respiratory symptoms compared with participants in households with no quantified concentrations. HCHO exposure and frequent incense exposure were associated with increased neurologic symptoms.

Our results provide a sound scientific foundation from which to design future studies. A more refined assessment of incense exposure and potential health effects is needed, given the ubiquitous nature of the exposure. We recommend incense burning be considered a significant source of particulate and gaseous pollutants in future indoor air and health studies in regions of the world where incense is commonly used. Additional exploration of indoor air pollutant concentrations and associated health effects, for example of HCHO and neurologic symptoms, is warranted. Future research should investigate how the infiltration of outdoor air pollutants contributes to indoor air pollutant concentrations. In addition, we recommend further investigation of temporal and spatial variation in indoor air pollutants, their sources, and potential health effects. With a better understanding of the factors and sources contributing to increased indoor air pollutants, steps can be taken to control and reduce pollutant concentration exposures in this population and globally.

## Supplemental Material

(8.9 MB) PDF
