# Supplementary material for: Indoor Air Pollutants and Health in the United Arab Emirates
Source: Environ Health Perspect. 2012 Feb 22;120(5):687–94. doi: 10.1289/ehp.1104090 (PMC3346777; doi:10.1289/ehp.1104090)

Supplemental Material

Indoor Air and Health in the United Arab Emirates (UAE)

Karin B. Yeatts[1*], Mohamed El-Sadig[2], David Leith[3], William Kalsbeek[4], Fatma Al-Maskari[2], David Couper[5], William E. Funk[6], Taoufik Zoubeidi[2], Ronna L. Chan[1], Chris B.Trent[3], Christopher A. Davidson[3], Maryanne G. Boundy[3], Maamoon M. Kassab[7], Mohamed Y. Hasan[2], Ivan Rusyn[3], Jacqueline MacDonald Gibson[3], Andrew F. Olshan[1].

TABLES

Supplemental Table 1. Size and Density of National Population by Emirate…………………...3

Supplemental Table 2. Comparison of UAE 2005 Census and Participating

Study Households, by Emirate ……………………………………………………………...4

Supplemental Table 3. Correlations among PM and Gas Concentrations ……..………………..5

Supplemental Table 4. Frequency of Other Respiratory Symptoms in Study Participants………6

Supplemental Table 5. Frequency of Neurologic Symptoms in Study Participants.………..…..7

Supplemental Table 6. Adjusted prevalence odds ratios of respiratory symptoms and doctor-diagnosed asthma associated with quantified household SO_2, NO_2, H_2S, formaldehyde concentrations …………………………………………………………………………….8

Supplemental Table 7. Adjusted prevalence odds ratios of neurologic symptoms associated with quantified household formaldehyde concentrations and daily incense burning ………………….9

FIGURES

Supplemental Figure 1. Respiratory Symptoms and Indoor Sources of Combustion (Tobacco Smoking, Gas Stoves in Attached Kitchens, Gas Stoves, Incense)……………………………..12

Supplemental Figure 2. Respiratory Symptoms and Indoor $PM_{2.5,}$ $PM_{c\,,}$ $PM_{10,}$ and CO…........13

Supplemental Table 1. Size and Density of National Population by Emirate

Emirate	UAE Census 2005 National Population	Area, km^2 from UAE official site, http://www.government.ae/web/guest/seven-emirates	National Population Density 2005*
Abu Dhabi	350,277	67,340	5
Dubai	137,573	3,885	35
Al Sharjah	138,272	2,590	53
Ajman	39,231	259	151
Um al Quaim	15,873	777	20
Ras al Khaimah	87,848	1,684	52
Fujairah	56,421	1,165	48
UAE Total	825,495	77,700	11

*population per km^2

Supplemental Table 2. Comparison of UAE 2005 Census and Participating Study Households, by Emirate

	UAE Census 2005 National Population	UAE 2005 Census of nationals, percent*	Indoor Air and Health Study HH level, number	Indoor Air and Health Study HH level, percent*
Abu Dhabi	350,277	42	248	40
Dubai	137,573	17	120	19
Al Sharjah	138,272	17	90	14
Ajman	39,231	5	34	5
Um al Quaim	15,873	2	22	4
Ras al Khaimah	87,848	11	68	11
Fujairah	56,421	7	45	7
Total N	825,495		627	

* Chi-square test of goodness-of-fit p-value=0.074, comparing national emirate percentages with UAE Indoor Air and Health percentages.

Supplemental Table 3. Correlations among PM and gas concentrations.

		**Indoor SO_2**	**Indoor NO_2**	**Indoor H_2S**	**Indoor CO**	**Indoor HCHO**
Indoor PM_{10}	Pearson's Correlation Coefficient	0.012	0.29	-0.003	-0.067	0.024
	p-value	0.77	0.49	0.94	0.11	0.56
	Number of observations	573	574	574	573	574
Indoor PM_c	Pearson's Correlation Coefficient	0.28	0.041	0.0041	-0.057	0.038
	p-value	0.50	0.33	0.92	0.18	0.36
	Number of observations	573	574	574	573	574
Indoor $PM_{2.5}$	Pearson's Correlation Coefficient	-0.05	-0.02	-0.03	-0.09	-0.03
	p-value	0.26	0.61	0.50	0.04	0.47
	Number of observations	573	574	574	573	574

Supplemental Table 4. Frequency of Other Respiratory Symptoms in Study Participants

**Respiratory Symptoms**[a]	*Value*	*N*[b]	*Wtd %* [c]	*95% CI*[d]
Dry cough at night, not from cold	Yes	272	19.09	[17.2, 21.0]
	No	1303	79.66	[77.7, 81.6]
	Missing	15	1.26	[0.7, 1.8]
Sinus infection in last 12 months	Yes	195	11.85	[10.3, 13.4]
	No	1379	86.81	[85.1, 88.5]
	Missing	16	1.34	[0.8, 1.9]
Shortness of breath in last 12 months	Yes	199	13.63	[11.9, 15.3]
	No	1376	85.12	[83.4, 86.9]
	Missing	15	1.26	[0.7, 1.8]
Shortness of breath at least once per month	Yes	126	8.27	[6.9, 9.6]
	No	70	4.98	[3.9, 6.0]
	Missing	1394	86.75	[85.1, 88.4]
Difficulty breathing/chest tightness in last 12 months	Yes	172	11.99	[10.4, 13.6]
	No	1400	86.47	[84.8, 88.1]
	Missing	18	1.55	[0.9, 2.2]
Difficulty breathing/chest tightness at least once per month	Yes	109	7.02	[5.8, 8.3]
	No	62	4.94	[3.9, 6.0]
	Missing	1419	88.04	[86.4, 89.6]
Cough in last 12 months	Yes	320	23.49	[21.4, 25.6]
	No	1255	75.26	[73.1, 77.4]
	Missing	15	1.26	[0.7, 1.8]
Cough at least once per month	Yes	177	12.60	[11.0, 14.2]
	No	143	10.89	[9.4, 12.4]
	Missing	1270	76.51	[74.4, 78.6]

[a] Includes adults, adolescents, children, [b] N = Number of Individuals, [c] Wtd% = **Percentages weighted by participant-level sampling weights [d] 95% Confidence Intervals

Supplemental Table 5. Frequency of Neurologic Symptoms in Study Participants[a]

**Neurologic Symptoms**	*Value*	*N*[b]	*Wtd %* [c]	*95% CI*[d]
Headache in last 12 months	Yes	641	46.48	[44.0, 48.9]
	No	933	52.25	[49.8, 54.7]
	Missing	16	1.26	[0.7, 1.8]
Headache at least once per month	Yes	413	27.26	[25.1, 29.4]
	No	226	19.05	[17.1, 21.0]
	Missing	951	53.69	[51.2, 56.1]
Difficulty concentrating in last 12 month	Yes	241	15.79	[14.0, 17.6]
	No	1331	82.54	[80.7, 84.4]
	Missing	18	1.67	[1.0, 2.3]
Difficulty concentrating at least once per month	Yes	162	10.90	[9.4, 12.4]
	No	76	4.47	[3.5, 5.5]
	Missing	1352	84.63	[82.9, 86.4]
Forgetfulness in last 12 months	Yes	353	25.34	[23.2, 27.5]
	No	1217	72.99	[70.8, 75.2]
	Missing	20	1.66	[1.0, 2.3]
Forgetfulness at least once per month	Yes	248	18.04	[16.1, 19.9]
	No	102	7.22	[6.0, 8.5]
	Missing	1240	74.74	[72.6, 76.9]
Dizziness in last 12 months	Yes	180	11.86	[10.3, 13.4]
	No	1394	86.86	[85.2, 88.5]
	Missing	16	1.29	[0.7, 1.8]
Dizziness at least once per month	Yes	101	5.93	[4.8, 7.1]
	No	78	5.92	[4.8, 7.1]
	Missing	1411	88.15	[86.6, 89.7]

[a] Includes adults, adolescents, children, [b] N = Number of individuals, [c] Percentages weighted by participant-level sampling weights [d] 95% Confidence Intervals

Supplemental Table 6. Adjusted prevalence odds ratios of respiratory symptoms and doctor-diagnosed asthma associated with quantified household SO_2, NO_2, H_2S, and formaldehyde concentrations.[a]

Respiratory Outcomes	SO_2		NO_2		H_2S		HCHO	
	OR	95%CI	OR	95%CI	OR	95%CI	OR	95%CI
Ever having wheezing and whistling in the chest	1.79	1.05-3.05	1.63	0.84-3.16	1.91	0.96-3.78	1.31	0.71-2.42
Wheezing in last 12 months	1.03	0.34-3.09	2.48	0.62-9.93	0.66	0.19-2.32	0.64	0.21-1.98
Wheezing in last 4 weeks	4.63	1.33-16.19	4.04	0.90-18.21	6.03	1.00-36.26	3.48	0.81-14.89
Wheezing limited speech to 1 or 2 words between breaths	3.53	1.06-11.74	3.48	0.99-12.22	6.85	1.31-35.81	4.18	1.23-14.22
Ever doctor diagnosed asthma	1.95	1.13-3.36	2.34	1.11-4.93	1.90	1.00-3.60	1.32	0.73-2.37
Had dry cough at night, not from cold	1.31	0.80-2.14	1.90	1.00-3.60	1.70	0.93-3.08	1.56	0.98-2.49
Sinus infection in last 12 months	1.25	0.71-2.18	1.77	1.07-2.94	1.76	0.89-3.48	1.49	0.84-2.64
Shortness of breath in last 12 months	1.26	0.76-2.10	1.88	0.84-4.22	1.46	0.66-3.26	1.55	0.97-2.48
Shortness of breath one or more times a month	2.61	0.75-9.12	1.07	0.20-5.66	1.32	0.32-5.50	3.68	1.11-12.27
Difficulty breathing/chest tightness in last 12 months	0.97	0.55-1.70	1.46	0.60-3.60	1.36	0.57-3.23	1.43	0.83-2.46
Difficulty breathing/chest tightness one or more times a month	4.34	1.71-11.05	4.70	1.58-13.98	3.27	1.20-8.91	6.52	1.91-22.31
Cough in last 12 months	0.66	0.41-1.09	1.25	0.66-2.38	1.27	0.77-2.10	1.02	0.65-1.59
Cough one or more times a month	4.08	1.80-9.29	3.03	1.10-8.35	2.55	1.04-6.27	3.59	1.70-7.55

[a] Models adjusted for gender, urban/rural area, age group, and household tobacco smoke exposure.

Supplemental Table 7. Adjusted prevalence odds ratios of neurologic symptoms associated with quantified household formaldehyde concentrations, and daily incense burning.[a,b]

Neurologic Symptoms	HCHO		Daily Incense Burning	
	OR	95%CI	OR	95%CI
Headache in last 12 mos	1.14	0.75-1.74	1.87	1.09-3.21
Headache at least once per mo	2.57	1.11-5.97	2.05	1.22-3.45
Difficulty concentrating in last 12 mos	1.47	1.02-2.13	3.08	1.70-5.58
Difficulty concentrating at least once per mo	2.15	0.86-5.37	1.08	0.21-5.60
Forgetfulness in last 12 mos	1.17	0.71-1.90	2.68	1.47-4.89
Forgetfulness at least once per mo	1.22	0.61-2.46	2.64	1.02-6.78
Dizziness in last 12 mos	1.64	0.97-2.77	1.41	0.48-4.15
Dizziness at least once per mo	5.85	2.35-14.56	3.06	0.79-11.90

[a] Models adjusted for gender, urban/rural area, age group, and household tobacco smoke exposure. [b] Daily incense burning compared with burning incense once a week or less.

Supplemental Figure 1. Respiratory Symptoms and Indoor Sources of Combustion (Tobacco Smoking, Gas Stoves in Attached Kitchens, Gas Stoves, Incense)

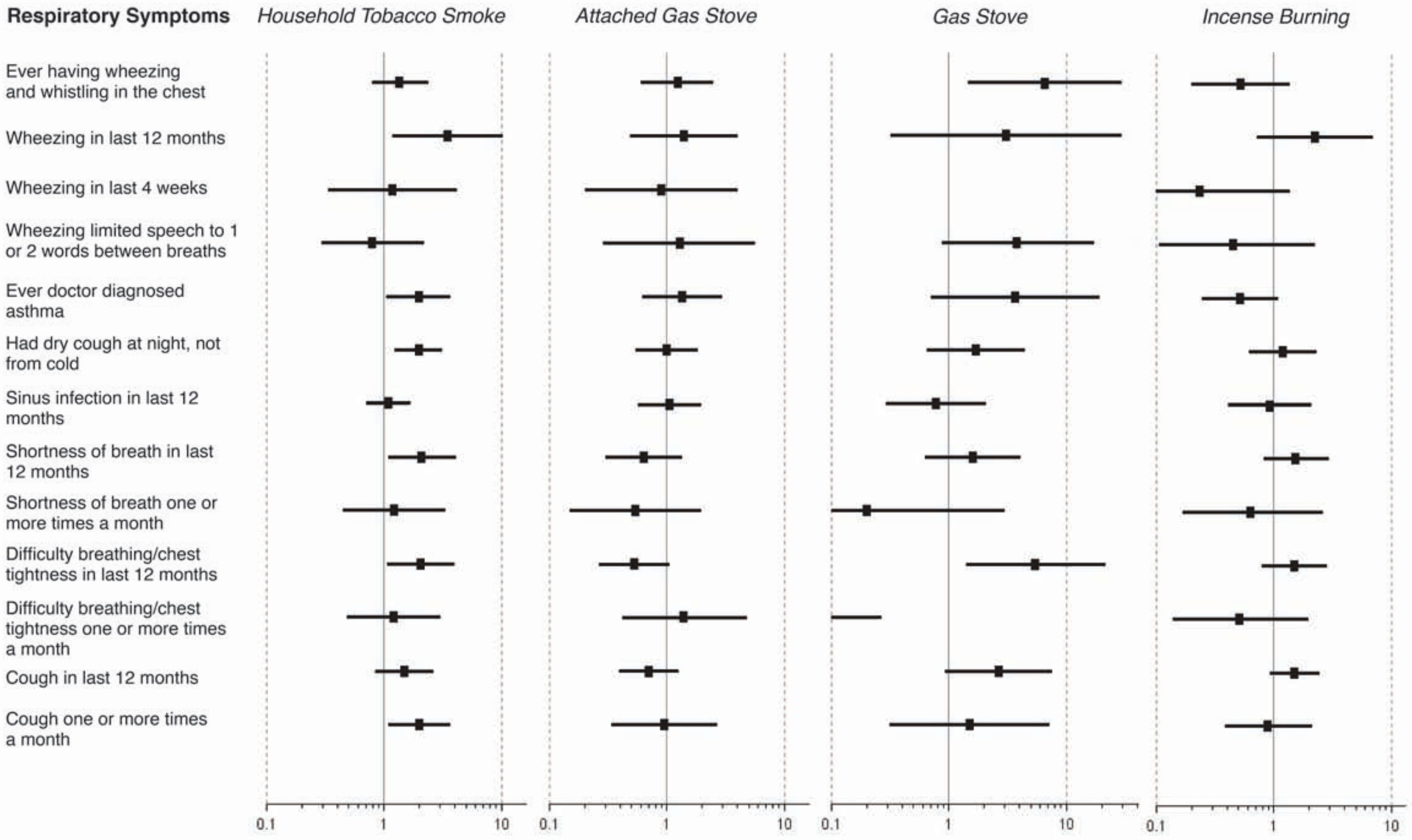

Supplemental Figure 2. Respiratory Symptoms and Indoor $PM_{2.5}$, PM_C, PM_{10}, and CO

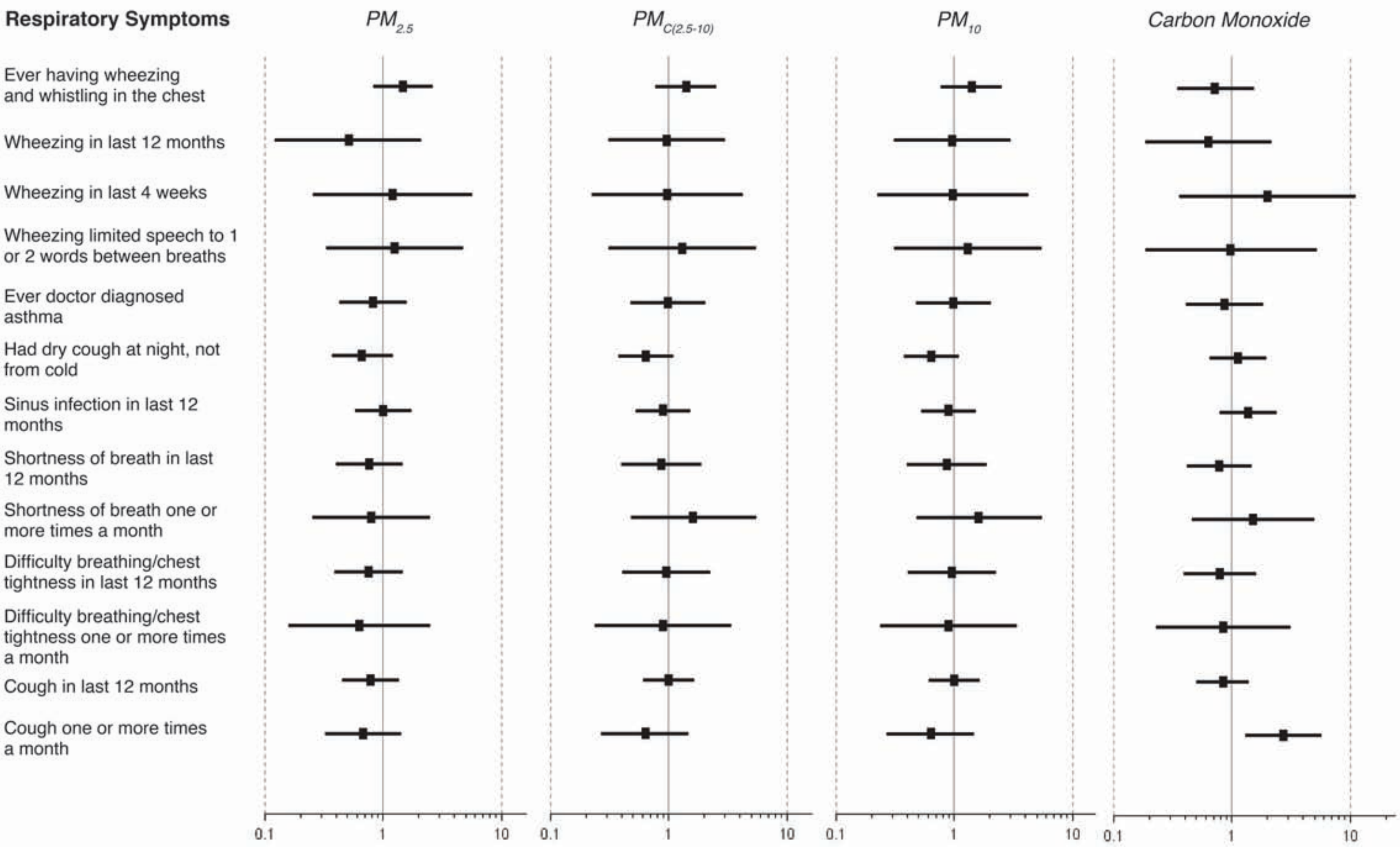

Supplement: (8.9 MB) PDF [file ehp.1104090.s001.pdf]
